# Thermo-Responsive Polyurethane Hydrogels Based on Poly(ε-caprolactone) Diol and Amphiphilic Polylactide-Poly(Ethylene Glycol) Block Copolymers

**DOI:** 10.3390/polym8070252

**Published:** 2016-07-05

**Authors:** Shan-hui Hsu, Cheng-Wei Chen, Kun-Che Hung, Yi-Chun Tsai, Suming Li

**Affiliations:** 1 Institute of Polymer Science and Engineering, National Taiwan University, Taipei 10617, Taiwan; r02549010@ntu.edu.tw (C.-W.C.); d00549005@ntu.edu.tw (K.-C.H.); z02232142@gmail.com (Y.-C.T.); 2 Institut Europeen des Membranes, UMR CNRS 5635, Universite de Montpellier, Place Eugene Bataillon, 34095 Montpellier Cedex 5, France; lisuming@hotmail.com

**Keywords:** waterborne polyurethane, poly(ε-caprolactone), polylactide–poly(ethylene glycol), small angle X-ray scattering (SAXS), hydrogel

## Abstract

Waterborne polyurethane (PU) based on poly(ε-caprolactone) (PCL) diol and an amphiphilic polylactide-poly(ethylene glycol) (PLA-PEG) diblock copolymer was synthesized. The molar ratio of PCL/PLA-PEG was 9:1 with different PLA chain lengths. The PU nanoparticles were characterized by dynamic light scattering (DLS), small angle X-ray scattering (SAXS) and rheological analysis. The water contact angle measurement, infrared spectroscopy, wide angle X-ray scattering (WAXS), thermal and mechanical analyses were conducted on PU films. Significant changes in physio-chemical properties were observed for PUs containing 10 mol % of amphiphilic blocks. The water contact angle was reduced to 12°–13°, and the degree of crystallinity was 5%–10%. The PU dispersions underwent sol-gel transition upon the temperature rise to 37 °C. The gelation time increased as the PLA chain length increased. In addition, the fractal dimension of each gel was close to that of a percolation cluster. Moreover, PU4 with a solid content of 26% could support the proliferation of human mesenchymal stem cells (hMSCs). Therefore, thermo-responsive hydrogels with tunable properties are promising injectable materials for cell or drug delivery.

## 1. Introduction

Polyurethanes (PUs) are polymers mainly consisting of long chain oligodiol as the soft segment and diisocyanate as the hard segment, in which short chain diol or diamine is added, serving as a chain extender. Conventional synthesis methods involve organic solvents, leading to concerns of volatile organic compounds [1]. In contrast, the process of synthesizing waterborne PU is more eco-friendly, and therefore, water-based PUs are more favorable compared to conventional PUs. The commonly-applied approach to uniformly disperse PU particles in an aqueous solution is the incorporation of an ionic chain extender into the chemical structure of PU [2].

Amphiphilic copolymers, which contain both hydrophilic and hydrophobic parts, have drawn much attention for biomedical applications in the past few decades. One category of these block copolymers consists of polylactide and poly(ethylene glycol) (PEG). PLA presents outstanding biodegradability and biocompatibility, high mechanical strength and plasticity and is widely used in surgical repair, drug delivery and tissue engineering [3]. PEG is highly hydrophilic and biocompatible and provokes less immune responses [4]. Furthermore, PEG can reduce protein adsorption onto PLA segments [4,5]. In addition, PLA-PEG copolymers present better biodegradability than the PLA homopolymers [6]. Therefore, copolymers combining PEG and PLA are promising for many biomedical applications, in particular as drug carrier [7]. PEG is more likely to move close to the surface of the material since it has relatively lower surface energy. This surface rearrangement is obvious especially in aqueous media [5]. Amphiphilic PLA-PEG copolymers would self-assemble to form micelles once placed in an aqueous environment. PLA segments are located in the interior of micelles, while PEG segments are at the surface. The solution of micelles may respond to the change of temperature, and gelation can occur. By changing the ratio of PLA to PEG, various kinds of environment responsive materials can be synthesized [8].

Hydrogels responding to a changing environment can be used as drug or cell delivery carriers, scaffolds for tissue engineering and wound dressings [9,10,11]. The cell-laden hydrogels have been used in regenerative medicine. However, some cells, such as mesenchymal stem cells, require adhesion onto the hydrogels to survive. Therefore, the viability and proliferation of MSCs need to be evaluated when the material is used for tissue regeneration [12]. Among these, thermo-responsive hydrogels appear the most promising. In other words, a polymer solution at room temperature turns into a hydrogel after injection into the human body without adding toxic curing agents [13]. Common examples are copolymers of PEG and poly(propylene glycol) (PPG) and poly(N-isopropylacrylamide) (PNIPAAm), which form gels when heated. However, PNIPAAm is not degradable in vivo [14]. Hence, the focus of research moves to copolymers composed of PEG, PLA, poly(lactide-*co*-glycolide) (PLGA) or poly(ε-caprolactone) (PCL) [15]. Thermo-responsive gelation requires that the polymer be amphiphilic and have an appropriate hydrophilic hydrophobic balance. Because most of these copolymers are linear, in order to make them mechanically strong enough to be applied in the fields of biomedicine, higher molecular weight is preferred. Nonetheless, the length of PEG has a significant influence on the gelation temperature. The concentration at which the gelation occurs decreases as the portion of hydrophobic blocks increases, whereas the gelation temperature rises as the molecular weight of the hydrophobic blocks increases [16].

In this study, PUs are synthesized, which consisted of PCL as the main soft segment. Part of the soft segment structure (10%) is replaced with PLA-PEG diblock copolymers with different PLA block lengths to improve the hydrophilicity. By using dynamic light scattering (DLS) and transmission electron microscopy (TEM), the particle size and the surface charge were determined. The gelation properties were investigated through rheological analyses. Fourier transform-infrared spectroscopy (FTIR) and wide-angle X-ray diffraction (XRD) were performed to evaluate hydrogen bonding and crystallinity changes in order to elucidate the influencing factors on gelation. By combining the above analyses, we could determine how the PLA block length of PLA-PEG copolymers affects the thermo-responsive gelation properties of the PU nanoparticle dispersion.

## 2. Materials and Methods

### 2.1. Synthesis of PLA-PEG Block Copolymer

Four PLA-PEG diblock copolymers were synthesized by ring opening polymerization of l-lactide using monomethoxy poly(ethylene glycol) (mPEG), as listed in Table 1. l-lactide and mPEG (*M*_n_ = 2000) were supplied by Purac and Sigma, respectively. L-lactide and mPEG were first added into a flask at different molar ratios. Then, the catalyst zinc lactate (0.1 wt %) was added. The flask was degassed and sealed under vacuum, and the polymerization was conducted at 140 °C. After 3 days, the product was dissolved in dichloromethane (CH_2_Cl_2_), precipitated in diethyl ether and further dried under vacuum [17]. The PLA-PEG diblock copolymers were named LA-EO diblock diol.

### 2.2. Synthesis of Waterborne PU

**Figure 1:**
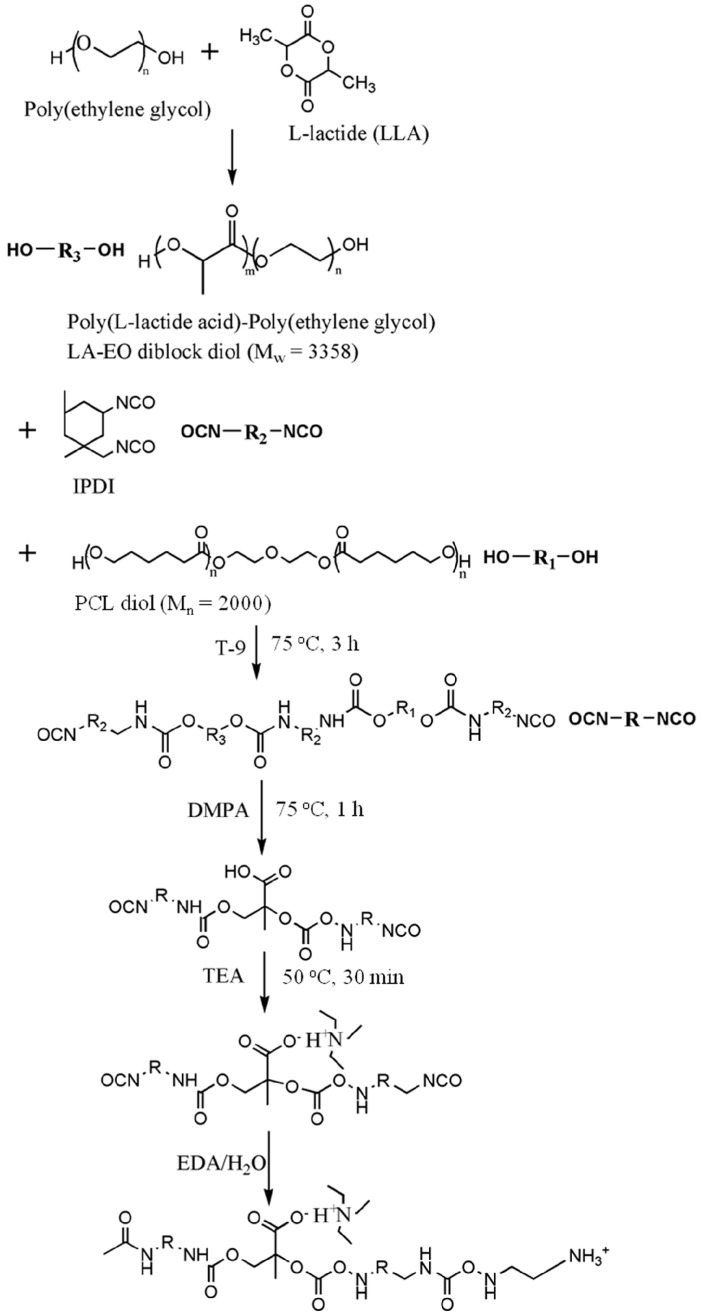
The synthesis process for PU with the LA-EO diblock diol as a part of the soft segment. IPDI, isophorone diisocyanate; DMPA, 2,2-bis(hydroxymethyl)propionic acid; TEA, triethylamine; EDA, ethylenediamine.

PU was prepared from a two-step reaction process previously described [18]. The reaction scheme is shown in Figure 1. The reactants were isophorone diisocyanate (IPDI, from Evonik Degussa GmbH, Essen, Nordrhein-Westfalen, Germany), ethylenediamine (EDA, from Tedia, Fairfield, OH, USA), 2,2-bis(hydroxymethyl)propionic acid (DMPA, from Sigma Aldrich, St. Louis, MI, USA), triethylamine (TEA, from RDH, Spring Valley, CA, USA), methyl ethyl ketone (MEK, from J.T. Baker, Phillipsburg, NJ, USA), and T-9. The reaction was performed in a 250-mL round-bottomed four-necked separable flask with a mechanical stirrer, a thermometer and a condenser with a drying tube and N_2_ inlet. The flask was immersed in an oil bath in order to achieve a constant-temperature environment. First, PCL diol (*M*_n_ = 2000 from Sigma Aldrich, St. Louis, MI, USA) and LA-EO diblock diol were added into the dried flask, followed by the addition of IPDI and T-9. The solution was stirred at 105 °C for 3 h. DMPA and MEK were then added with the temperature controlled at around 70 °C, and the solution was stirred for 1 h. The heater was turned off, and after the temperature of the mixture dropped to 50 °C, TEA was added and the mixture stirred for 30 min. An EDA solution in deionized water was then added, and the mixture was vigorously stirred for another 30 min. The stoichiometric ratio of IPDI, oligodiols, DMPA, EDA and TEA was 3.52:1:1:1.52:1. Residual MEK and TEA were removed by vacuum distillation. The obtained PU dispersion in water had a solid content of about 30%. The PU dispersion was either directly characterized or was cast onto Teflon plates to prepare films for further bulk characterization. All PU dispersions were characterized or cast in 3 days after synthesis to avoid aggregation.

### 2.3. Physico-Chemical Characterization

The PU dispersion was diluted with distilled water to bring down the solid content to 0.3 wt %. The hydrodynamic diameter (*D*_h_) and zeta potential of the PU nanoparticles were measured by the DelsaTM Nano C Particle Analyzer (Beckman Coulter, Brea, CA, USA).

The water contact angle of the PU film surface was measured by a contact angle analyzer (FTA-1000 B, First Ten Angstrom Company, Portsmouth, VI, USA) at room temperature. Water droplets were around 5 μL in volume for each measurement. Values of water contact angles were obtained at various spots on the film and averaged. The attenuated total reflection-infrared (ATR-IR) spectroscopy was used to characterize the surface chemistry of the films. The spectra between wavenumbers of 4000 and 650 cm^−1^ were collected by using a spectrophotometer (Spectrum 100, Perkin-Elmer, Waltham, MA, USA).

The tensile mechanical properties were determined by a universal testing instrument (HT-8504, Hung Ta Co., Nantun District, Taichung, Taiwan) following the ASTM standard protocol (ASTMD638.10). The samples were tailored into a size of 25 mm (length) × 5 mm (width) × 1 mm (thickness), and the stretching rate was 100 mm·min^−1^.

The thermogravimetric analysis (TGA) was conducted with a thermogravimetric analyzer (TA Instrument, New Castle, DE, USA). Each sample (5 mg) was placed in an alumina cubicle, heated at a rate of 10 °C per min under N2. Differential scanning calorimetry (DSC, Pyris 6, Perkin-Elmer, Waltham, MA, USA) was used to determine the glass transition temperature (*T*_g_) and the melting temperature (*T*_m_). The heating rate was 10 °C per min, and the measurement was operated in a temperature range of −70–150 °C. The weight average molecular weight was estimated by gel permeation chromatography (GPC, Waters, Milford, MA, USA) in dimethylacetamide using polystyrene standards.

XRD was used to examine the crystallization characteristics of the PU films. The equipment was operated at a power of 2 kW, and measurements were made in the scattering angle range of 10°–30°. The degree of crystallinity was calculated from the integration area under characteristic peaks.

For a better understanding of the microstructure of PU nanoparticles, SAXS was conducted at the beamline 23A of National Synchrotron Radiation Research Center (Hsinchu Science Park, Hsinchu, Taiwan). The photon energy of about 10 eV was applied. According to the Guinier method [ln(I) = ln(I_0_) − *q*^2^*R*_g_^2^/3], by plotting the logarithm of the intensity versus the square of scattering vector, the fractal dimension (df) was obtained from the negative value of slope of the plot. The radius of gyration (*R*_g_) was estimated under the circumstance that *qR*_g_ < 1.3.

The rheological properties of the PU dispersions were measured in 3 days after synthesis. Storage beyond 2 weeks could significantly shorten the gelation time and increase the gel modulus. The rheological analysis was carried out using a rheometer (RS-5, TA Instruments, New Castle, DE, USA) with a cone and plate geometry at a frequency of 1 Hz and a deformation of 1%. The time-dependent dynamic storage modulus (*G*’) and loss modulus (*G*’’) were measured for each PU. The point where *G*’ = *G*’’ was defined as the point of sol-gel transition.

### 2.4. Cell Culture

Human umbilical cord-derived mesenchymal stem cells (hMSCs) obtained from BIONET Corp (Neihu District, Taipei, Taiwan) were cultured in T150 tissue-culture flasks (Falcon, BD Bioscience, San Jose, CA, USA). The culture medium was composed of low-glucose Dulbecco’s Modified Eagle Medium (LG-DMEM), 10% fetal bovine serum (FBS; SAFC Biosciences, St. Louis, MI, USA), 1% penicillin-streptomycin (Caisson, Logan, UT, USA) and 0.4% gentamicin (Gibco, Waltham, MA, USA). The incubation of the cells took place in a humidified incubator with 5% CO_2_ at 37 °C. Cells of the eleventh to thirteenth passage were used in the analyses.

### 2.5. Cell Labeling

Before being mixed with the PU4 dispersion with a solid content of 26% and gelation at 37 °C, the cells were stained with a red fluorescent dye PKH26 (Sigma-Aldrich, St. Louis, MI, USA). The dye could be stably introduced into the cell membrane, thus labeling the cells. Cells with a density of 2 × 10^6^ cells/mL were mixed with PKH26 with a concentration of 2 × 10^−6^ M, and the labeling was stopped with the complete medium. Lastly, the cells were washed to be ready for use.

### 2.6. Cell Viability and Vitality

Cell viability was measured by staining the cells in the dispersion with Solution 5, which contained propidium iodide (PI) and acridine orange, marking the dead cells and serving as a counterstain, respectively, and a patented fluorophore VB-48™, which showed fluorescence only when bound to reduced thiols, could be used to analyze the cell health condition. Therefore, the distribution of live cells and the intensity of thiol levels in cells could be obtained using the NucleoCounter^®^ NC-3000™ system (ChemoMetec, Davis, CA, USA). Specifically, the reduced thiol type in the cells was glutathione (GSH), whose decrease in concentration could be related to apoptosis. Thus, the lower fluorescent intensity, which indicating the lower level of GSH, implied the reduced healthiness of the cells. The healthiness of hMSCs was compared to those in culture medium with or without dimethyl sulfoxide (DMSO, 10 wt %).

### 2.7. Cell Proliferation

The MTT assay was applied to measure cell proliferation. The cell-laden hydrogels were washed in phosphate-buffered saline (PBS), followed by the addition of 200 μL of 0.1 mg/mL tetrazolium dye (Sigma-Aldrich, St. Louis, MI, USA) and incubation for 4 h at 37 °C. After incubation, the tetrazolium dye was removed. The reduced product of tetrazole, the purple formazan, was dissolved by DMSO and detected at 570 nm using a microplate reader (SpectraMax^®^ M5, Molecular Devices LLC, Sunnyvale, CA, USA). The optical density was normalized to that measured for cells (cell density: 2 × 10^6^ cells/mL) before mixing with hydrogels (100% viability). The viabilities were measured at the 1st, 2nd, 3rd and 7th day of culture. The medium without cells and cells in a blank well (tissue culture polystyrene (TCPS)) were used as control groups, where the MTT assay was performed for cells on 24-well plates (cell density: 3 × 10^4^ cells/wells), and the viabilities were normalized to those measured for cells before the culture. The results were proven to be reproducible in at least three independent experiments. Through one-way analysis of variance, *p*-values < 0.05 indicated significant statistical differences.

## 3. Results

### 3.1. Characterization of PU Nanoparticles and PU Films

The SAXS profiles of PUs with different PLA chain lengths are displayed in Supplemental Figures S1–S3. The size, surface charge and molecular weight of each type of PU nanoparticle are listed in Table 1. PU0, which only consisted of PCL as the soft segment, exhibits a hydrodynamic diameter (*D*_h_) of 39.3 nm. With the presence of 10% of PLA-PEG as the soft segment, the *D*_h_ of PU decreased to 27–32 nm. The NPs of PU2 and PU4 were of bigger sizes, and the former had the largest *D*_h_ of 32 nm, while the NPs of PU1 and PU3 had smaller and close values of *D*_h_ around 27–28 nm. The NPs of PU1 and PU3 had higher *D*_g_/*D*_h_ values of 1.17 and 1.15, respectively. The NPs of PU2 and PU4 had very similar *D*_g_/*D*_h_ values around 1.09. On the other hand, PU0 nanoparticles had a zeta potential of −57 mV. Replacing 10% of the PCL soft segment with the PLA-PEG increased the zeta potential of PU nanoparticles to −34–−29 mV, demonstrating that introduction of amphiphilic blocks reduced the surface charge. Since each nanoparticle comprises several polymer chains, by dividing the molecular weight of a single nanoparticle with the molecular weight of a single polymer chain, the number of polymer chains in one PU nanoparticle was estimated. The obtained numbers of polymer chains were 322, 105, 94, 192 and 131 for PU0–PU4, respectively.

**Figure 2:**
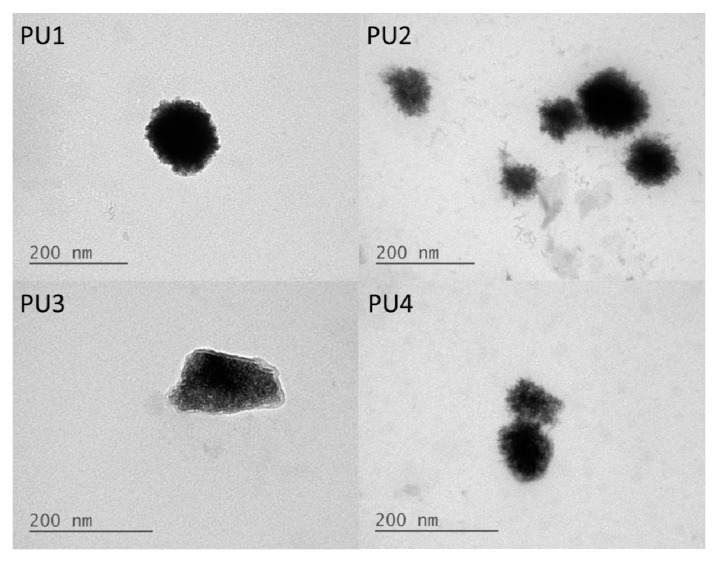
TEM images of PU1, PU2, PU3 and PU4 in the aqueous dispersions.

The TEM images of PU nanoparticles are shown in Figure 2. It was observed that the core of the nanoparticle was covered by a relatively brighter shell layer, which might be the more hydrophilic PEG segment.

The contact angle values of the films prepared from various types of PUs are listed in Table S1. PU0 presents a contact angle of 83°. In contrast, the contact angles of PU1–PU4 were approximately in the range of 12°–13°, i.e., much lower than that of PU0. This finding suggests that PU films containing PLA-PEG are much more hydrophilic.

The ATR-IR spectra of different types of PUs are displayed in Supplemental Figure S4. The characteristic peaks of NCO groups at 2260–2280 cm^−1^ and OH groups at 3200–2600 cm^−1^ were not detected for all PUs, confirming that all monomers reacted completely. The characteristic peaks of amide bonding in PU at 3350 cm^−1^ (N–H groups) and 1730 cm^−1^ (C=O groups) were observed. The absorption peaks of C–O–C groups were observed at 1060–1250 cm^−1^. The peak at 1100 cm^−1^ was ascribed to the ether group of PEG in PU, while the peaks at 2890 cm^−1^ were attributed to stretching of CH and CH_3_ in PLA and the CH_2_ groups in PCL and PEG.

### 3.2. Rheological Analyses

**Figure 3:**
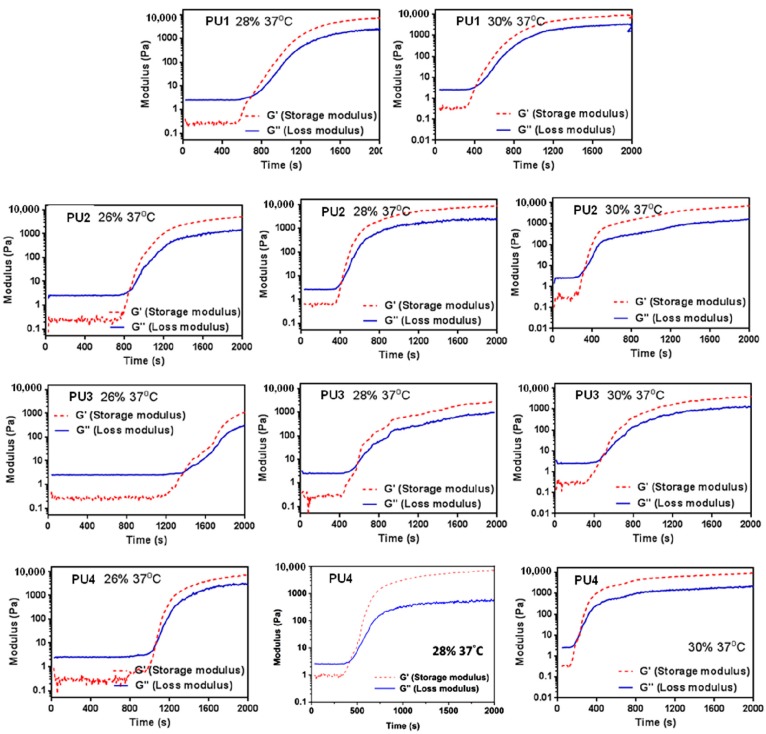
Time-dependent changes of the storage modulus (*G*’) and loss modulus (*G*’’) of PU1, PU2, PU3 and PU4 of different total solid contents after the temperature rises from 25–37 °C.

The rheological profiles of PU NP dispersions upon the temperature rise to 37 °C are demonstrated in Figure 3. The time-dependent gel strength for PU NP dispersions with different PLA chain lengths and different solid contents (wt %) are detailed in Table 2. The gelation rate of PU at 37 °C decreased as the PLA chain length increased. At 26 wt %, as the PLA chain length increased, the gelation time also increased and reached its maximum at PU3, then decreased for PU4. At 28 or 30 wt %, the relation between gelation time and PLA chain length became insignificant. The gelation time was obviously shorter as the solid content of PU dispersions increased. The gel strength for each PU also increased significantly with time. PU1 and PU2, at 28 wt % and after 30 min in 37 °C, had a gel strength of more than 6000 Pa. In contrast, PU3 with a longer PLA chain length had much lower gel strength. As for PU4, it had a relatively higher gel strength at 30 wt % and after 20 min upon 37 °C.

### 3.3. Fractal Dimension of PU Gel

**Figure 4:**
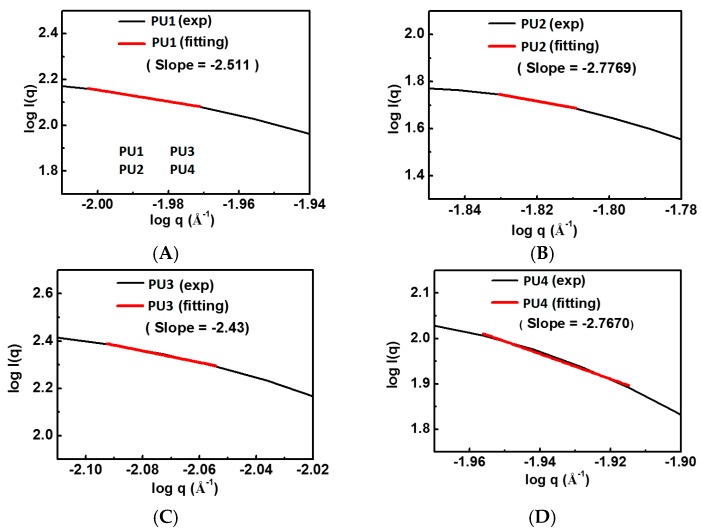
SAXS profiles of various PU dispersions at 25 °C: (**A**) PU1; (**B**) PU2; (**C**) PU3; and (**D**) PU4.

The SAXS profiles of the PU dispersions are displayed in Figure 4. The fractal dimensions of various types of PUs obtained from the slopes of the curves at 37 °C are listed in Supplemental Table S2. PU2 and PU4 had close and larger fractal dimensions of 2.78 and 2.77, respectively, while PU1 and PU3 had relatively smaller values of 2.51 and 2.43.

### 3.4. Mechanical and Thermal Properties of PU Films

The tensile stress-strain curves of various PU films are shown in Supplemental Figure S5 and from which the obtained Young’s modulus, 100% modulus, tensile strength and elongation at break are listed in Table 3. After replacing a part of the PCL soft segment of PU with the PLA-PEG block, the Young’s modulus and tensile strength of PU films decreased. Among the PUs containing PLA-PEG blocks, PU2 and PU4 had the larger Young’s modulus and tensile strength. In particular, PU4 had a tensile strength of around 33 MPa, which was the closest to that of PU0. On the other hand, replacing a part of the PCL soft segment with the PLA-PEG block tended to increase the elongation of the PU films to more than 600%.

TGA curves of different types of PU films are shown in Supplemental Figure S6. The onset decomposition temperature (*T*_onset_), thermal decomposition temperature (*T*_d_) and glass transition temperature (*T*_g_) are listed in Table 3. Among the PUs, PU1 had the lowest *T*_onset_, and PU4 had the lowest *T*_d_. PU3 had the highest *T*_onset_ and *T*_d_. PU1-4 all had a higher *T*_g_ than PU0. However, *T*_g_ seemed to decrease as the LA chain length increased.

**Figure 5:**
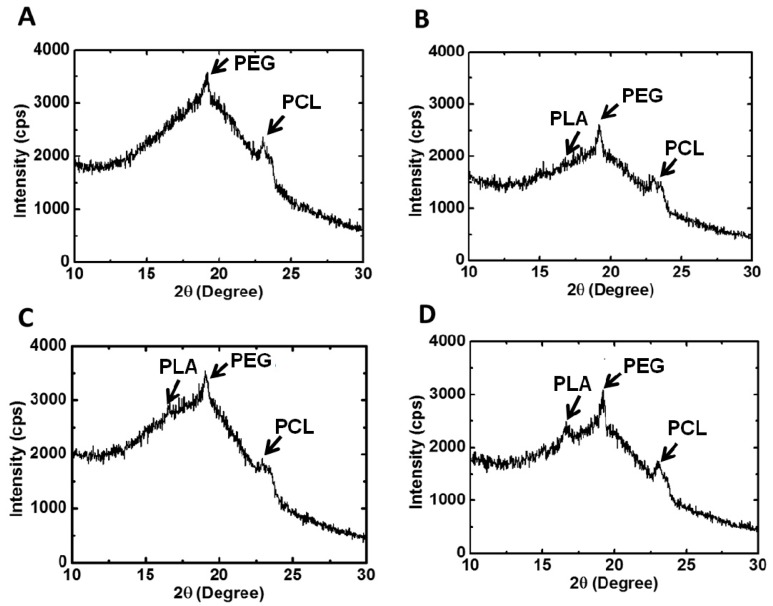
X-ray diffraction patterns and the degree of crystallinity of PU films: (**A**) PU1, (**B**) PU2, (**C**) PU3 and (**D**) PU4.

The XRD profiles and the calculated degree of crystallinity of various types of PU films are both displayed in Figure 5. PU0 had a broad band without any characteristic diffraction peak, implying that it was amorphous. On the other hand, PU1-4 showed different characteristic peaks of crystallization. Peaks at 2θ = 16.7° and 2θ = 19.2° were associated with PLA and PEG blocks, respectively.

Peaks at 2θ = 21.3° and 2θ = 23.5° were attributed to the crystallization of the PCL segment. PU2 had the largest degree of crystallization, and PU4 had the second largest one. In comparison, PU1 and PU3 had close and smaller values of the degree of crystallization. The crystallization of PU1-3 was mainly associated with the PEG block and PCL, whereas in PU4, the PLA block contributed a fair amount of crystallization, as much as the PEG block and PCL segment.

### 3.5. Cell Viability and Vitality

**Figure 6:**
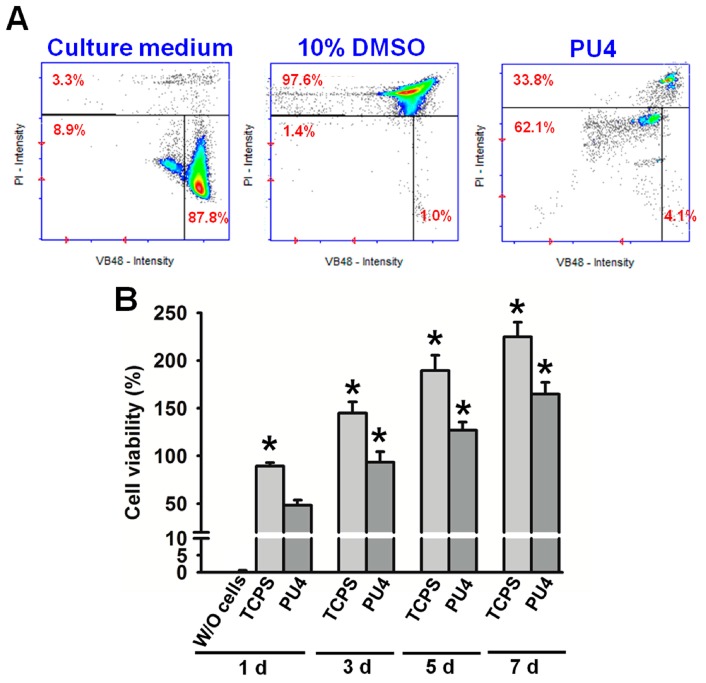
(**A**) The cell vitality (%) of hMSCs in PU4 hydrogel and in the culture medium with or without 10 wt % of DMSO; (**B**) the cell viability (%) of hMSCs in PU4 hydrogel and in the blank well (tissue culture polystyrene TCPS) at the first, third, fifth and seventh day of culture. The values were normalized to those of cells before culture (100% viability). Significance (*p* < 0.05): * higher than the PU4 group at 1 day. “W/O” indicates the medium without cells.

Results from cell viability and vitality tests are displayed in Figure 6. The analysis for cells in PU4 hydrogel (26% solid content) using the NucleoCounter^®^ NC-3000™ system showed that the number of live cells (viable cells) upon initial contact with the hydrogel was about 66.2%. The number of dead cells was about 33.8%. Out of the living cells, 4% of them were not affected by the presence of the hydrogel. Most cells (62%) showed a reduction of healthiness right after contact with the hydrogel. On the other hand, the cells cultured in the medium showed a viability of 96.7% with 87.8% of them being healthy. In contrast, 97.6% of the cells were dead in the medium containing 10 wt % DMSO.

### 3.6. Cell Proliferation

**Figure 7:**
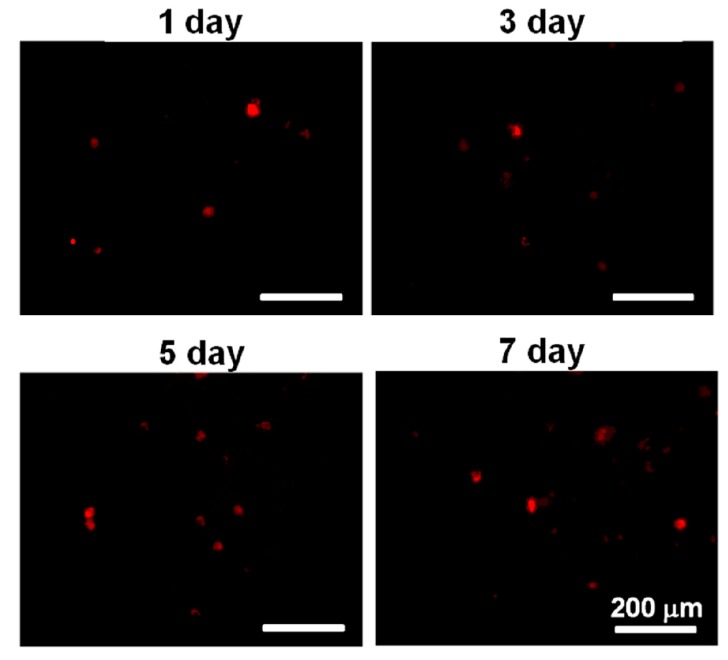
The images of PKH26-stained cells in PU4 hydrogel at the first, third, fifth and seventh day of culture.

Although most of the live cells reduced their health condition upon the initial contact with the PU4 hydrogel, they showed significant proliferation after being cultured for three days and continued to proliferate at the seventh day of culture, as shown in Figure 6 and Figure 7. The optical density of the MTT assay of the medium without cells was very low. The cell viability in PU4 hydrogel returned to 100% after three days and reached 170% after seven days, compared to that of the initially seeded cells.

## 4. Discussion

All of the PU nanoparticles in the study had very small hydrodynamic diameters and negative zeta potentials, demonstrating that they were stably dispersed in aqueous solutions. The negative charges on the surfaces of the particles may be attributed to the COO^−^ functional group on the hard segment. The hydrodynamic diameters and diameters of gyration of the PU nanoparticles with amphiphilic PLA-PEG blocks decreased while the zeta potentials increased. The decrease in nanoparticle size might be due to the tight package of the PLA blocks. The increase in zeta potentials was probably a result of surface enrichment of the PEG segment. Based on the ratio of *D*_g_ over *D*_h_, which was 0.775 for spherical particles and 1.54 for random coils [19], PU2 and PU4 were more spherical than PU1 and PU3.

By dividing the molecular weight of a single nanoparticle by the molecular weight of a single polymer chain, the number of polymer chains in one PU nanoparticle was estimated. The number of polymer chains in a single PU nanoparticle was reduced significantly in all PU nanoparticles containing various PLA-PEG blocks (<200 polymer chains in one nanoparticle), compared to the PCL-based PU (>300 polymer chains in one nanoparticle).

The contact angles of the PU films containing various PLA-PEG blocks were much lower than the PCL-based PU, indicating that the surface was much more hydrophilic. The changes in contact angles might be associated with the more hydrophilic PEG blocks in the structure [20]. Furthermore, the TEM images showed that the PU nanoparticles were covered in a relatively brighter layer of presumably the PEG block. These data were consistent with the less negative zeta potential.

The ATR-IR spectra of PU did not demonstrate any peaks around 2260–2280 cm^−1^ (–NCO) or 3200–3600 cm^−1^ (–OH), indicating that all monomers reacted completely. On the other hand, the absorption peaks showed up around 1060–1250 cm^−1^ in PUs containing PLA-PEG blocks, which were associated with the PEG segment. The appearance of this band agreed with the greater hydrophilicity of the surface containing the amphiphilic blocks.

All PU NP dispersions in this study had low viscosities below 37 °C and therefore could be mixed well with cells. Upon the temperature rise to 37 °C, gelation occurred in a short period of time (~min) in 30% dispersions. At a lower solid content (26%), as the PLA chain length increased, the gelation time also increased. This could be attributed to the decreased PCL content, leading to less hydrogen bonding and smaller interaction between NPs [21], preventing the coacervation of PU NPs. On the other hand, when the solid content was 28% or 30%, the gelation time of PU NP dispersions became shorter, and the particles were aggregated more readily. The gel modulus (*G*’) increased with time at 37 °C. Besides, PU1, PU2 and PU4 gels at higher solid contents had large *G*’. In contrast, PU3 had a significantly lower G’ compared to the other gels, which could be associated with the lower tendency of the crystallization of the sample [22]. The obtained fractal dimension from the rheological analysis was around 2.4–2.8 for each gel, which is close to the one (~2.5) calculated from the theory of the percolation cluster. Moreover, the commonly-used biological polymer gelatin also has a value of the fractal dimension of 2.5 [23]. All PLA-PEG block-containing PUs (PU1–PU4) showed relatively good gel strength at 37 °C without any crosslinking agent.

The gelation of PU hydrogels was related to the interaction between soft segments, demonstrated by their crystallization [24]. However, the crystallization of wet hydrogels was difficult to detect due to the high water content. Therefore, PU emulsion deprived of water was used to prepare PU films, in which the degree of crystallization was measured. With the incorporation of PLA-PEG blocks into PU, the steric hindrance tended to affect the arrangement of polymer chains, causing the amorphous PCL-based PU to crystallize. The PLA blocks could facilitate the crystallization of PEG and PCL segments [25]. Nonetheless, as the LA chain length increased to a certain degree, namely in PU3 and PU4, the effect became less obvious. As a result, the degree of crystallinity reached a maximum at PU2 and then decreased. Judging from the crystallization peaks in XRD, the crystallization of PU1, PU2 and PU3 was mainly attributed to PEG and PCL segments, while PLA blocks contributed a fair amount of crystallization in PU4 in comparison with PEG and PCL segments.

To investigate whether the crystallization affected the mechanical properties accordingly, the tensile properties of PU films were analyzed. For the PU films, the decrease of tensile strength in PUs containing PLA-PEG blocks might be associated with the lower PCL content of the PUs. PCL is more likely to form hydrogen bonds between polymer chains, and thus, a lower PCL content is linked to lower tensile strength [24]. PU2 and PU4 films had relatively larger tensile strength and Young’s modulus, which may be ascribed to the higher degree of crystallinity. The elongation of PUs containing PLA-PEG blocks increased significantly to over 600%, suggesting the more elastic behavior of the resulting PU films.

Regarding the thermal properties, the *T*_g_ of the PU films was mainly attributed to the crystallization of PLA blocks [26]. The pyrolytic temperatures *T*_onset_ and *T*_d_ both increased as the PLA chain lengths increased, but decreased abruptly in PU4, indicating that excessive PLA could make the polymer susceptible to decomposition.

The viability of hMSCs upon the initial contact with the PU4 hydrogel was only around 66%, and the health condition of most live cells was affected by the presence of hydrogel. However, the cells proliferated significantly after three days of culture, and the growth continued at the seventh day of culture. The cell viability reached 170% of the initially seeded cells. These data indicated that the hydrogel could support the proliferation of hMSCS regardless of the negative influence on the cell health at the early stage of culture.

In summary, the nanoparticle dispersions of PUs containing PLA-PEG blocks could be stably dispersed in aqueous solutions due to the hydrophilicity of the NPs. Upon the temperature rise to 37 °C, gelation occurred quickly in all samples. The fractal dimension of each PU gel was close to 2.5, similar to that of a percolation cluster. Taken together, these materials were fast thermos-responsive hydrogels with tunable gel strength and properties. Furthermore, PU4 with a solid content of 26% could support the proliferation of hMSCs.

## 5. Conclusions

In this study, four types of waterborne PUs based on 90% PCL diol and 10% amphiphilic PLA-PEG blocks were successfully synthesized. The prepared PUs, PU1–PU4, showed significantly different properties from PU0 consisting of 100% PCL as soft segments. The surface of the films of PU1–PU4 became much more hydrophilic. The ATR-IR spectra and TEM images both demonstrated that the surface was enriched in PEG blocks. The degree of crystallinity varied from 5%–10% depending on the PLA chain length. The dispersions of PU1–PU4 had low viscosities at room temperature, but upon the temperature rise to 37 °C, gelation occurred with storage modulus *G*’ > 1 kPa. The gelation time depended on the PLA chain lengths and the solid contents. The fractal dimensions were close to that of a percolation cluster and commonly-used biological polymer gelatin. These PU dispersions were thermo-responsive, and their properties could be adjusted by changing the PLA chain length. Among the dispersion studied, PU4 (10 mol % LA22EO45 in the soft segment) demonstrated fast gelation and a strong gel modulus (>100 Pa) in 10 min at solid content ≥28%. In addition, PU4 with a solid content of 26% could support the proliferation of hMSCs, making it promising for use as an injectable cell delivery vehicle.

## Figures and Tables

**Table:** 

PU films	PLA (2θ = 16.7°)	PEG (2θ = 19.2°)	PCL (2θ = 23.5°)	Total
PU0	NA	NA	0	0
PU1	NA	2.38	2.92	5.30
PU2	0.38	4.44	5.66	10.48
PU3	0.64	2.32	2.19	5.15
PU4	2.94	3.11	2.92	8.97

**Table 1:** The soft segment composition, hydrodynamic diameter (*D*_h_), diameter of gyration (*D*_g_), zeta potential and molecular weight of polyurethane (PU) NPs. PCL, poly(ε-caprolactone).

PU Abbreviation	LA-EO Diblock type	Molar percent of the PU soft segment (%)			*D*_h_ (nm)	Zeta potential (mV)	*D*_g_ (nm)	*M*_w_, NP	*D*_g_/*D*_h_
		PCL	PLA	PEG					
PU0	–	100	0	0	39.30 ± 0.9	−57.00 ± 2.1	40.0 ± 0.0	4.20 × 10^7^	1.08
PU1	LA_10_EO_45_	90	1.82	8.18	27.20 ± 1.0	−32.08 ± 0.4	32.0 ± 0.2	1.01 × 10^7^	1.17
PU2	LA_12_EO_45_	90	2.10	7.90	32.35 ± 0.6	−33.47 ± 0.7	35.3 ± 0.4	9.86 × 10^7^	1.09
PU3	LA_19_EO_45_	90	2.97	7.03	28.65 ± 0.2	−29.25 ± 0.7	33.2 ± 0.5	1.13 × 10^7^	1.15
PU4	LA_22_EO_45_	90	3.28	6.72	30.60 ± 1.4	−34.63 ± 0.6	33.5 ± 0.2	1.24 × 10^7^	1.09

**Table 2:** The gelation time and time-dependent gel strength of PU dispersions.

PU dispersions	Total solid content	Gelation time (s) as *G*’ = *G*’’	Gel strength (G′, Pa)		
			10 min	20 min	30 min
PU1	26%	NA	NA	NA	NA
	28%	694	0.79	1,344	6,529
	30%	403	110.3	4,788	8,515
PU2	26%	845	0.278	1,068	4,382
	28%	532	41	3,779	6,370
	30%	316	837	5,357	7,944
PU3	26%	1,378	0.2531	0.331	377.8
	28%	580	18.5	758	2,365
	30%	499	56.49	1,607	3,589
PU4	26%	1,052	0.2248	520	5,320
	28%	523	187	3,650	5,490
	30%	229	2,336	6,435	8,606

**Table 3:** The mechanical, thermal properties of PU films.

PU films	Young’s modulus (MPa)	100% Modulus (MPa)	Tensile strength (MPa)	Elongation at break (%)	*T*_onset_ (°C)	*T*_d_ (°C)	*T*_g_ (°C)	*T*_m_ (°C)
PU0	30.9 ± 7.9	5.30 ± 0.1	34.9 ± 3.1	535.5 ± 19	266	372	−53	NA
PU1	13.6 ± 3.7	4.26 ± 1.0	21.8 ± 2.0	634.6 ± 0.1	224	321	−48	56
PU2	17.5 ± 2.2	4.59 ± 0.5	29.5 ± 4.1	636.7 ± 0.7	226	320	−49	57
PU3	14.1 ± 1.1	4.12 ± 0.2	24.7 ± 1.7	636.9 ± 0.8	236	343	−52	61
PU4	18.8 ± 1.8	4.84 ± 0.2	33.1 ± 2.7	637.0 ± 0.1	226	316	−50	56

## Supplementary Materials

Supplementary Materials can be found at www.mdpi.com/2073-4360/8/7/252/s1.
