# Supplementary material for: Thermo-Responsive Polyurethane Hydrogels Based on Poly(ε-caprolactone) Diol and Amphiphilic Polylactide-Poly(Ethylene Glycol) Block Copolymers
Source: Polymers (Basel). 2016 Jul 5;8(7):252. doi: 10.3390/polym8070252 (PMC6432218; doi:10.3390/polym8070252)
Supplement: Supplementary file 1 [file polymers-08-00252-s001.pdf]

Supplementary Materials: Thermo-Responsive Polyurethane Hydrogels Based on Poly(ε-caprolactone) Diol and Amphiphilic Polylactide-Poly(Ethylene Glycol) Block Copolymers

Shan-hui Hsu, Cheng-Wei Chen, Kun-Che Hung, Yi-Chun Tsai and Suming Li

Table S1. The water contact angle of PU films

PU films	Water contact angle (°)
PU0	83.0 ± 1.8
PU1	13.9 ± 3.5
PU2	13.3 ± 5.8
PU3	13.0 ± 5.9

Table S2. Molecular weight, complex viscosity and fractal dimension of PU.

PU abbreviation	Molecular weight	Complex vi scosity (Dispel rsion, cP)	Fractal dim ension (D_f)
PU0	130,411	2.16	NA
PU1	104,703	2.59	2.51
PU2	105,072	2.21	2.78
PU3	58,603	2.32	2.43
PU4	94,473	2.63	2.77

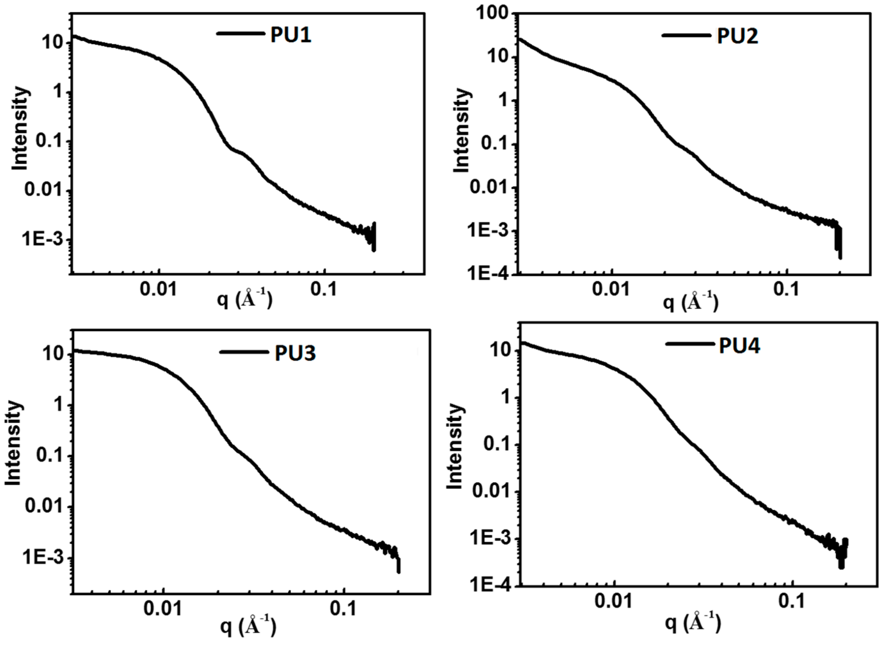

Figure S1. Origin plot.

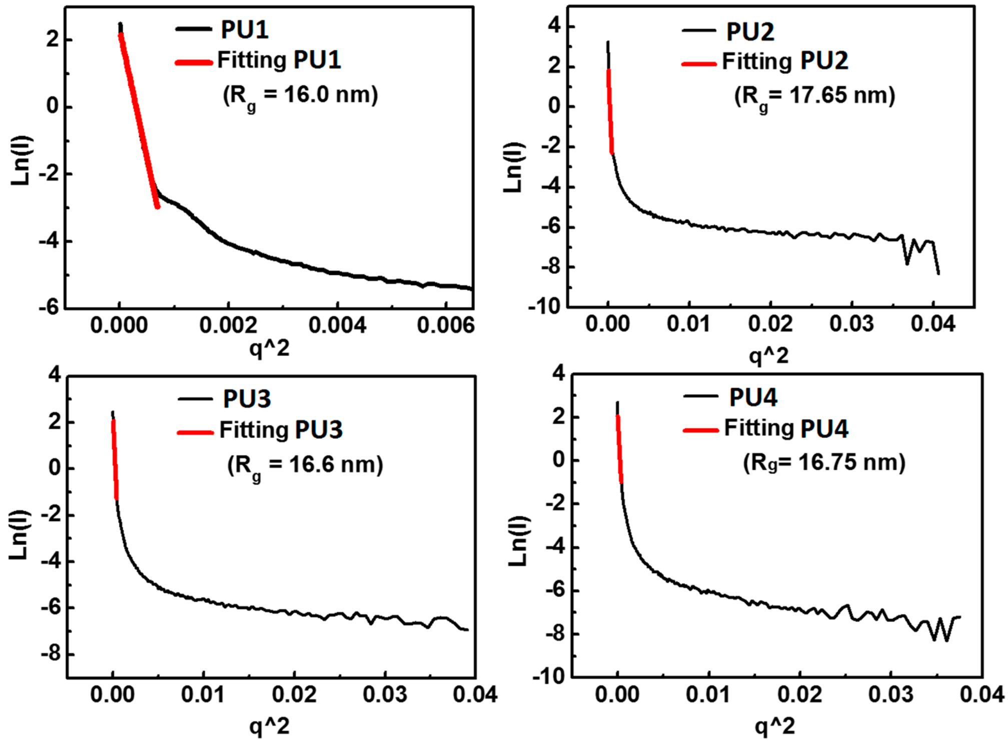

Figure S2. Guinier plot.

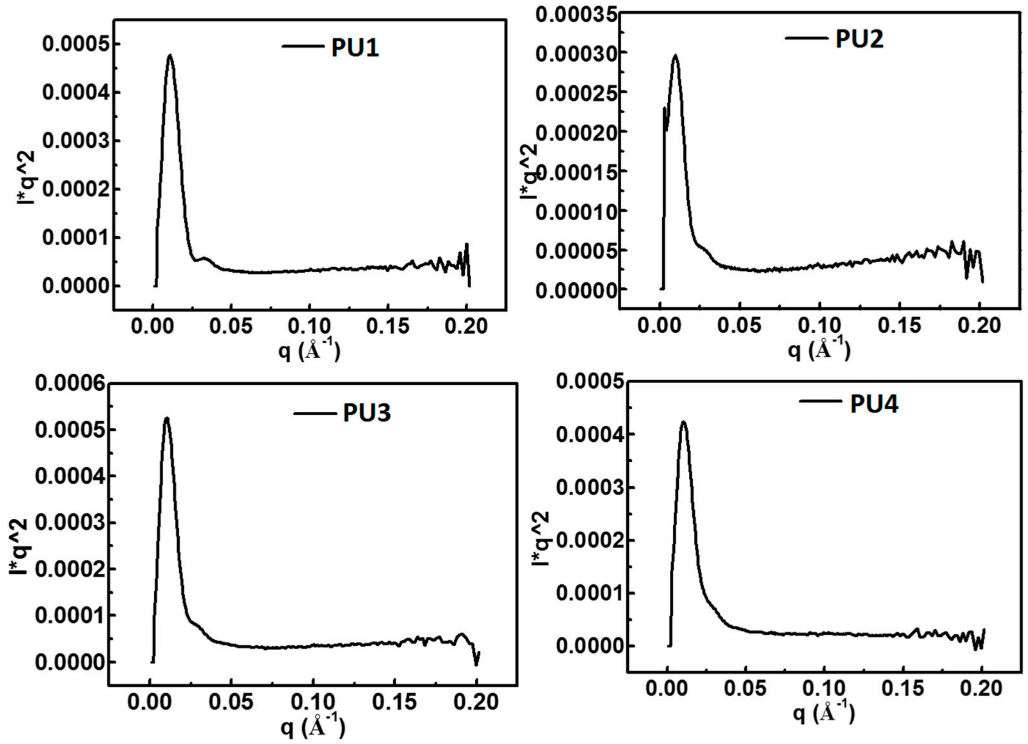

Figure S3. Kratky plot.

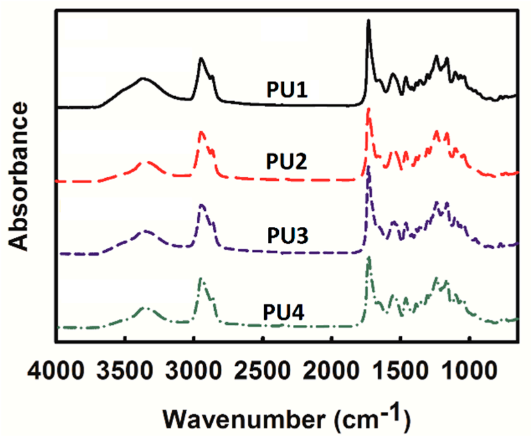

Figure S4. FT-IR spectra of the PU.

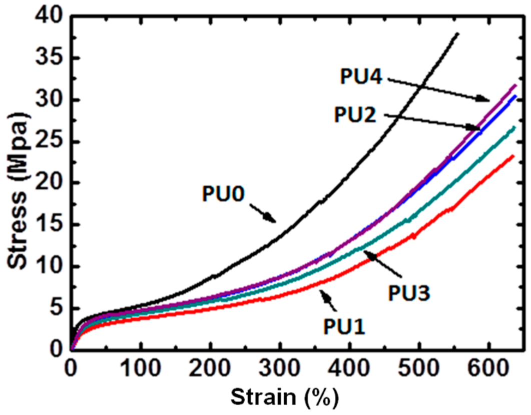

Figure S5. Stress-strain curve.

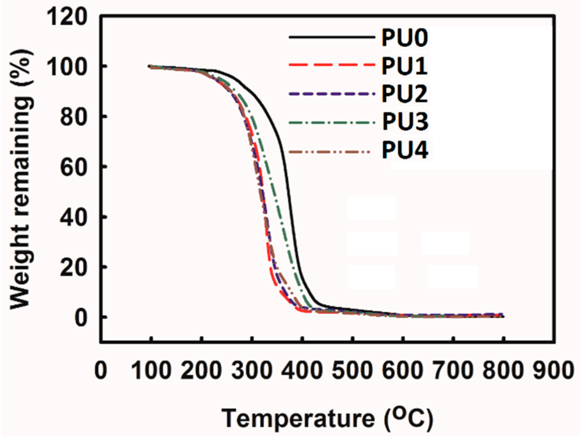

Figure S6. TGA curves.

© 2016 by the authors; licensee MDPI, Basel, Switzerland. This article is an open access article distributed under the terms and conditions of the Creative Commons Attribution (CC-BY) license (http://creativecommons.org/licenses/by/4.0/).
